# Molecular diagnosis of putative Stargardt disease probands by exome sequencing

**DOI:** 10.1186/1471-2350-13-67

**Published:** 2012-08-03

**Authors:** Samuel P Strom, Yong-Qing Gao, Ariadna Martinez, Carolina Ortube, Zugen Chen, Stanley F Nelson, Steven Nusinowitz, Deborah B Farber, Michael B Gorin

**Affiliations:** 1 Jules Stein Eye Institute, University of California Los Angeles, 200 Stein Plaza, Los Angeles, CA, 90095, USA; 2 Department of Human Genetics, University of California Los Angeles, 695 Charles E. Young Drive South, Los Angeles, CA, 90095, USA

**Keywords:** Stargardt Disease, Macular Degeneration, Exome, Mutation Screening, Molecular Diagnostics, *ABCA4*, *PRPH2*

## Abstract

**Background:**

The commonest genetic form of juvenile or early adult onset macular degeneration is Stargardt Disease (STGD) caused by recessive mutations in the gene *ABCA4*. However, high phenotypic and allelic heterogeneity and a small but non-trivial amount of locus heterogeneity currently impede conclusive molecular diagnosis in a significant proportion of cases.

**Methods:**

We performed whole exome sequencing (WES) of nine putative Stargardt Disease probands and searched for potentially disease-causing genetic variants in previously identified retinal or macular dystrophy genes. Follow-up dideoxy sequencing was performed for confirmation and to screen for mutations in an additional set of affected individuals lacking a definitive molecular diagnosis.

**Results:**

Whole exome sequencing revealed seven likely disease-causing variants across four genes, providing a confident genetic diagnosis in six previously uncharacterized participants. We identified four previously missed mutations in *ABCA4* across three individuals. Likely disease-causing mutations in *RDS/PRPH2, ELOVL,* and *CRB1* were also identified.

**Conclusions:**

Our findings highlight the enormous potential of whole exome sequencing in Stargardt Disease molecular diagnosis and research. WES adequately assayed all coding sequences and canonical splice sites of *ABCA4* in this study. Additionally, WES enables the identification of disease-related alleles in other genes. This work highlights the importance of collecting parental genetic material for WES testing as the current knowledge of human genome variation limits the determination of causality between identified variants and disease. While larger sample sizes are required to establish the precision and accuracy of this type of testing, this study supports WES for inherited early onset macular degeneration disorders as an alternative to standard mutation screening techniques.

## Background

Individuals with early-onset macular degeneration (MD) represent a diagnostic challenge. While observation of MD in a juvenile or young adult strongly implies an underlying genetic cause of disease, the spectrum of potential genes and mutations known to cause MD is extensive. Given the variability of clinical features that have been attributed to autosomal recessive mutations in the *ABCA4* gene [1-3], the most common clinical diagnosis for MD is Stargardt Disease [OMIM: #248200/#600110] (STGD). Rare cases of STGD or “Stargardt-like” disease phenotypes have been reported with mutations in *PRPH2*[4,5], *VMD2*[6]*, ELOVL4*[7-9] and *PROM1*[10]. These genes, as well as *ABCA4*, are also associated with clinically distinct phenotypes including retinitis pigmentosa, cone/rod dystrophy and pattern dystrophy. This complex arena of genes and clinical features complicates the nomenclature in this field; it is unclear how to classify individuals with classic Stargardt phenotype lacking recessive mutations in *ABCA4*. While we do not limit the term “retinitis pigmentosa” to a phenotype caused by mutations in a single gene, there are those who believe STGD should be restricted to only those cases caused by *ABCA4* mutations and “Stargardt-like” or juvenile macular dystrophy should be used for other genetic etiologies. For the purposes of this study, we classify our participants with early-onset macular degeneration as “putative Stargardt Disease” cases.

Since the initial identification of recessive mutations in the *ABCA4* gene in several large STGD pedigrees [3], a multitude of studies have attempted to assess the mutational spectrum of *ABCA4* in various ethnically restricted populations (Italy [11,12]; Spain [13,14]; Japan [15]; Portugal [16]; Hungary [17]; Denmark [18]). While each population appears to carry some founder mutations accounting for a significant proportion of disease alleles, hundreds of extremely rare or private variants have also been identified [19]. This allelic heterogeneity hampers the viability of standard panel-based mutation screening techniques and has lead to the creation of an *ABCA4* array designed to genotype over 600 individual DNA variants [19]. While this array is an effective screening tool, the high rate of private mutations in STGD prevents it from being a comprehensive solution.

Another factor making *ABCA4* such a difficult diagnostic target is its genomic structure. The large number of coding exons in *ABCA4* gene (50 coding exons resulting in a 6.8 kb mRNA [Refseq NM_000350]) makes PCR-based sequencing costly and time-consuming. Currently, the cost of PCR and dideoxy-based targeted *ABCA4* exon sequencing is approximately half that of WES. The costs of data analysis are difficult to compare, but WES analysis is highly automatable and can be performed in parallel as opposed to the highly labor-intensive analysis required to identify rare variants from individual sequence traces. Approaches using targeted next-generation sequencing hold great potential for *ABCA4* mutation screening [20,21], and may reduce the cost of single gene sequencing significantly.

Using a combination of methods to screen for *ABCA4* mutations in 121 patients from a heterogeneous patient population in our natural history study of Stargardt Disease, only half (59/121, 49%) of the cases were found to have two known or putative disease-causing *ABCA4* mutations on separate alleles. Nearly one third have a single mutation, and the remaining ~20% have no definite or probable disease-causing *ABCA4* mutations (manuscript in preparation). Highly sensitive next-generation targeted re-sequencing of *ABCA4* has been implemented on an independent sample set with similar results [20]. Whether individuals with putative Stargardt Disease with one or zero detected *ABCA4* mutations have non-traditional variation (e.g. promoter variants or epigenetic changes) missed by current mutation screening techniques or mutations in other genes is unclear at this time.

Providing a genetic diagnosis for individuals with putative Stargardt Disease is thus an ongoing challenge. Here we apply whole exome sequencing to nine putative STGD cases to assess the efficacy of this approach for molecular genetic diagnosis of this condition. All patients were previously screened and found to have zero (eight samples) or one (one sample) disease-causing mutation in *ABCA4*. While some of these probands express ocular imaging phenotypes not typically observed in classic STGD, all were diagnosed clinically as having STGD due to the early age at which signs of macular degeneration were initially identified.

In this study, we use the same WES methods and analytical tools as those implemented by laboratories now offering CLIA-approved, clinical WES. As such, we report these research findings as a preliminary surrogate for expected results from clinical WES of patients with retinal dystrophy.

## Methods

### Study Participants

Participant samples were recruited into this study under approval of the UCLA Institutional Review Board and consented prior to participation. Each was diagnosed by a referring retinal specialist and confirmed by a retinal expert at the Jules Stein Eye Institute, University of California Los Angeles (Gorin M.B) [22]. All research was conducted in accordance with the Health Insurance Portability and Accountability Act of 1996. A basic ophthalmologic exam, electroretinogram (ERG), optical coherence tomography (OCT) and imaging of the fundus was performed for each individual. Clinical phenotyping summaries can be found in Table 1. Subjects were selected for WES if they had an initial clinical diagnosis of Stargardt Disease and lacked mutations in *ABCA4* as screened by targeted dideoxy re-sequencing. One case, STGD-02, was included as a positive control having had one *ABCA4* mutation detected previously by Sanger sequencing.

**Table 1:** Clinical Information and Genetic Findings of Putative Stargardt Disease Cases

**Sample**	**Age Sex**	**Acuity OD Acuity OS**	**Clinical Notes**	**ERG Findings**	**Color vision**	***ABCA4*variants**	***PRPH2*variants**	**Other variants**
STGD-01	19 Male	20/400 20/160	Peripapillary sparing, discrete flecks; nummular atrophy	Normal rod Abnormal cone	No testing	p.N965S p.R2038W	.	.
STGD-02	15 Female	20/160 20/100	Few yellowish Flecks without autofluorescence	Normal rod Normal cone	Abnormal	p.Q636X p.G1961E*	.	.
STGD-03	58 Female	20/50 20/16	No significant atrophy	Abnormal rod Abnormal cone	No testing	.	p.W25C p.Q276X	.
STGD-04	51 Male	20/20 20/20	Peripapillary sparing both eyes; “stellate” pattern	Abnormal rod Abnormal cone	Abnormal	.	p.G167S	.
STGD-05	55 Female	20/25 20/20	Central atrophy with out-branching; "stellate" pattern	Normal rod Normal cone	Normal	.	p.T236X	.
STGD-06	34 Female	20/200 20/200	Classic Stargardt	Normal rod Abnormal cone	No testing	p.V989A	.	.
STGD-07	41 Male	20/40 20/640	Flecks, peripapillary sparing, central geographic atrophy	Normal rod Abnormal cone	No testing	.	.	.
STGD-08	37 Male	20/40 20/400	No peripapillary sparing/flecks; irregular geographic atrophy with RPE changes; thickening on OCT	Normal rod Normal cone	Abnormal	.	.	*CRB1* p.K801X
STGD-09	61 Male	20/25 20/25	"Horseshoe" pattern of atrophy	Normal rod Normal cone	No testing	.	.	*ELOVL4* p.A311T

“Age” is age at ascertainment. Abbreviations: ERG - Electroretinogram; RPE - Retinal pigment epithelium; OD - right eye; OS - left eye. * Variant previously observed by dideoxy sequencing. “Variants” indicates putatively causal variants and does not include predicted benign or common (polymorphic) variants.

### Whole Exome Sequencing Data Generation

Randomly fragmented genomic DNA libraries were created following standard protocols for high-throughput paired-end sequencing on the Illumina GA2x or HiSeq2000 instruments (Illumina Inc., San Diego, CA). The Agilent SureSelect 50 Mb capture kit was used to enrich the libraries for known coding loci in the human genome (Agilent Technologies; Santa Clara, CA). This kit has been shown to effectively capture >90% of these loci when an adequate average read depth is reached [23].

### Data Analysis

Nucleotide base calling and quality score assessment was performed using instrument-specific Real Time Analysis (RTA) software provided by Illumina. A combination of commercially or academically available tools and custom scripts were used to analyze the raw DNA sequence reads. Alignment to the human genome (hg19; NCBI build 37; Feb. 2009) was performed using Novoalign (Novocraft Technologies; Selangor, Malaysia). Merging, sorting, and other manipulation of aligned data was performed using SAMTools [24]. PCR clonal duplicate removal was performed using Picard http://picard.sourceforge.net. Quality score recalibration, genotyping, variant filtration, and coverage depth analysis were performed using the Genome Analysis Toolkit [25]. Variant consequence analysis was performed using SeattleSeq [26] which incorporates many databases including: “NCBI full genes”, dbSNP131, and the 1000 Genomes Project. Further analysis was performed using a combination of custom PERL scripts and Mathematica 7.0 (Wolfram Research; Champaign, IL).

Novel variants identified as putatively causal were screened for presence in the NHLBI Exome Sequencing Project database. Alleles present at ≥1% allele frequency in either sample population (European Caucasian or African American) were considered common alleles unlikely to contribute to rare disease, and thus removed from analysis.

### Additional Sequencing

Targeted amplification of *PRPH2* and *ELOVL4* coding sequences was performed using PCR (primer sequences available in Additional file 1 Supplemental Materials). PCR products were then sent out for dideoxy sequencing (Beckman Coulter; Brea, CA) and analyzed using Sequencher software (Gene Codes Corp.; Ann Arbor, MI).

## Results

### Exome sequencing completeness and quality

All libraries were successfully sequenced with a minimum mean on-target coverage depth of 44x (Additional file 2 Table S1). Samples sequenced on the Illumina HiSeq2000 had significantly higher coverage (average of 103x-121x) due to the increased volume of sequence reads produced by this instrument compared to the GA2x. Samples contained on average approximately 156,000 DNA variants from hg19, the vast majority of which overlap a known polymorphism (Additional file 2 Table S1). The two individuals of African American descent had an increased proportion of novel variants, consistent with the higher levels of genetic heterogeneity in that sub-population. Both the total number of variants and proportion of which overlap a known polymorphism were tightly correlated with read depth and are within the expected limits based on other studies [23].

Whole exome sequencing using the Agilent SureSelect 50 Mb capture kit provided high quality base calls for a minimum of 97.5% of the 6,822 base pair positions corresponding the translated *ABCA4* mRNA. For samples sequenced on the HiSeq2000 were covered, 100% of bases were covered ≥8x (Additional file 2 Table S1). Canonical splice acceptor and splice donor sites were captured at a similar rate.

### Individual Case Descriptions and Identified Variants

Clinical summaries, including visual acuity, age of recruitment, gender, ethnicity, and relevant ophthalmological findings are found in Table 1.

#### STGD-01

This participant exhibited several classic features of STGD (Table 1, Additional file 3 Figure S1). Panretinal cone dysfunction with preserved rod function was documented by ERG. Exome sequencing identified two missense variants (p.N965S and p.R2038W) previously reported as disease-causing in STGD [19].

#### STGD-02

This participant presented with atypical macular degeneration (Table 1, Additional file 4 Figure S2). Two rare coding variants in *ABCA4* were identified by exome sequencing in this participant. The p.G1961E missense variant is a known STGD mutation [19]. This variant was previously detected by PCR amplification and dideoxy sequencing and served as a positive control in this study. The second variant p.Q636X introduces a premature termination codon and is thus considered very likely deleterious.

#### STGD-03

This participant was documented to have central vision loss, more severe in the left eye (Table 1, Additional file 5 Figure S3a-S3b). Subretinal fluid was observed by OCT (Additional file 5 Figure S3b-S3c). Multifocal ERG showed near complete loss of the foveal peaks and uniformly reduced and delayed cone responses for the entire testing areas of both eyes. Two rare, novel coding variants in *PRPH2* (p.W25C and p.Q276X) were identified in this participant. As parental genetic material is not available and these two variants are non-adjacent, it is not possible to assess phase. The missense variant is predicted to be deleterious by all three algorithms implemented by Condel [27]and the nonsense variant is assumed to be deleterious.

#### STGD-04

This participant presented with bilateral symptoms including central vision distortion, light/dark adaption difficulty, and intermittent photopsias. Clinical findings were consistent with a pattern dystrophy (Table 1, Additional file 6 Figure S4). Full field and multifocal ERGs detected abnormal cone and rod amplitudes and implicit times. Color vision (FM100 Hue) was bilaterally abnormal, with greater severity for the left eye. A single rare coding variant in *PRPH2* was identified in this participant. This variant, p.G167S, has previously been linked to pattern dystrophy [28] and is predicted to be deleterious by Condel. The participant’s father’s vision became uncorrectable in the sixth decade of life, potentially consistent with a dominant mode of inheritance.

#### STGD-05

This participant showed signs of pattern dystrophy (Table 1, Additional file 7 Figure S5). A single rare nonsense variant (p.T236X) in *PRPH2* was identified in this participant. This variant has not been previously described, but is likely deleterious as it introduces a premature termination codon. The proband’s father and paternal grandfather suffered from night blindness but, by history, the visual impairment was not as severe as that of the proband.

#### STGD-06

This participant had classic features of STGD (Table 1, Additional file 8 Figure S6). The full field ERG showed abnormal cone responses with preserved rod function. Exome sequencing identified a single, previously described rare missense disease-causing variant in *ABCA4* (p.V989A). No other mutations were observed in any of the other genes known to cause retinal or macular dystrophies.

#### STGD-07

This participant had several classical STGD features including fundus flecks (Table 1, Additional file 9 Figure S7). The full field ERG showed abnormal cone responses with normal rod function. No clear candidate variants were found by exome sequencing in any of the known retinal or macular genes that might account for these clinical findings.

#### STGD-08

This participant was diagnosed with macular degeneration by age 16, but did not suffer from severe vision impairment until age 37. No fundus flecks were observable, though there was an irregular pattern of atrophy and RPE changes (Table 1, Additional file 10 Figure S8a-S8b). The central vision was impaired severely in the left eye but less so in the right. The optical coherence tomography study showed a diffuse thickening and loss of lamination of the central retina without the presence of cavitations or subretinal fluid (Additional file 10 Figure S8b-S8c). The retinal appearance, while atypical for patients with known Stargardt Disease, is similar to that seen in retinal dystrophies attributable to *CRB1*[29]. A novel nonsense mutation p.K801X in the developmental gene *CRB1* was found by exome sequencing. There is no family history of visual problems suggestive of a dominant mode of inheritance.

#### STGD-09

This participant presented with central vision loss and geographic atrophy in a perifoveal “horseshoe” pattern (Table 1, Additional file 11 Figure S9). Exome sequencing identified a novel missense variant in *ELOVL4*: p.A311T. An unconfirmed family history report indicated that his father may have had similar visual impairment.

### Additional *PHPR2* and *ELOVL4* sequencing

Direct re-sequencing of 68 additional putative Stargardt Disease cases identified five individuals with either of two previously reported *PRPH2* mutations (p.P210R [30] or p.172 W [5]). In total, rare coding variants in eight out of 77 (10.4%) putative STGD cases from six independent pedigrees were identified. This sample set is enriched for patients without two known *ABCA4* mutations, suggesting this pickup rate is likely elevated compared to the general STGD patient population. No additional individuals were found to harbor putative disease-causing variants in *ELOVL4*.

All eight individuals with at least one putatively deleterious *PRPH2* allele were over the age of 50 at ascertainment (age range 53–70 years). Two of these individuals also carry at least one disease mutation in *ABCA4* identified by direct sequencing. Three had severe atrophy, precluding the clinical observation of pattern dystrophy that may or may not have been present earlier in life. Among the five remaining cases, a clear pattern dystrophy can be appreciated in only four out of a possible ten maculae.

## Discussion

The most common cause of early-onset macular degeneration is Stargardt Disease (STGD). Even in the context of STGD - which is primarily caused by compound heterozygous missense, nonsense and/or splicing variants in *ABCA4* - there are key complicating factors that lead to problems in precise molecular diagnosis. In this study we examine the feasibility of performing whole exome sequencing on putative STGD cases as either a supplement or replacement for *ABCA4*-specific targeted methodologies. To do this, we sequenced genomic DNA from nine individuals with negative or unclear molecular diagnosis following direct sequencing of the major STGD disease gene *ABCA4*. The sample size of this study limits the determination of the general validity of this approach, but our results strongly suggest that mutation detection of STGD patients by WES is highly effective as compared to other methods.

Four out of five *ABCA4* mutations were not detected by PCR/dideoxy based exon sequencing and yet were detected in the context of whole exome sequencing in this study. Of the five total disease-causing *ABCA4* alleles found by WES, four (all except p.Q636X) have previously been described and are genotyped by the current ABCR array available from Asper Biotech, supporting the use of the genotyping array as a first-pass screening tool due to its low cost.

Two of these mutations were clearly visible as heterozygous variants upon re-analysis of the original sequencing traces. One of the newly identified mutations is visible on the original sequence chromatogram but is located within the first five bases of the read and thus indistinguishable from normal background noise. The chromatogram corresponding to the remaining variant had a high level of noise throughout. Other laboratories have noted similarly missed variants [31]. While currently considered the “gold standard” for molecular genetic diagnosis of SNV-type mutations, PCR-based dideoxy sequencing relies heavily on manual sequence trace inspection and is thus subject to human error. This method should and will continue to be relied upon for many applications, including mutation validation and genotyping of known alleles. However, it is difficult for a molecular diagnostic laboratory to maintain a high level of surveillance quality when performing a large number of these assays, as is required for mutation screening in STGD and other macular dystrophies.

Variant detection by exome sequencing is by comparison far more automated and less susceptible to this type of error. The sources of error in WES are systematic and readily measured. The major sources of erroneous findings by WES are insufficient or uneven read depth (“coverage”), mismapping, and incorrect variant annotation. While these sources of error are complex to resolve, they are highly consistent which has allowed computational advancements to dramatically improve results. In contrast, the confounding issues facing capillary gel electrophoresis-based dideoxy sequencing have remained relatively stable since the inception of this method. Our data supports the contention that WES provides reliable and efficient mutation detection for SNVs and indels in *ABCA4*, and is an attractive alternative to other methods.

Surprisingly, *RDS/PRPH2* mutations were identified by exome-seq in three individuals. This gene has been linked to several other retinopathies including pattern macular dystrophy [30,32-36] and retinitis pigmentosa [37], and it has been previously suggested that mutations in *PRPH2* can mimic the STGD phenotype [38] or modify disease severity [5]. However, *PRPH2* has not been considered a significant contributor to classic STGD. Indeed, two of the individuals carrying a heterozygous *PRPH2* mutation show a “stellate” pattern dystrophy in one eye. While this is consistent with previous reports of *PRPH2*-related disease [4,28,37-40], it is too subtle a finding to confidently sub-classify patients. The lack of concordance between two eyes of the same individual in both cases (Additional file 6 Figure S4 and Additional file 7 Figure S5) suggests incomplete expressivity of this endophenotype, suggesting that some cases with *PRPH2* related disease may show no identifiable signs of pattern dystrophy.

Additional mutations were detected by direct re-sequencing of *PRPH2* in a broader cohort of STGD cases. Complicating matters, two out of the eight people with rare missense or nonsense mutations in *PRPH2* also have mutations in *ABCA4*. One of these participants, a compound heterozygote for *ABCA4* mutations has two affected relatives who share the *PRPH2* mutations but neither of the *ABCA4* mutations. For these relatives, the *PRPH2* mutation appears to be disease-causing. In the proband with a single *ABCA4* mutation and a *PRPH2* mutation, the genetic etiology of the disease is less clear. At this time, it is not possible to assess the effect of this potentially digenic scenario, though it has been previously observed [5]. The presence of rare, coding *PRPH2* variants in STGD cases with one or more *ABCA4* mutations suggests that one must be cautious regarding attributing apparent cases of Stargardt Disease to *ABCA4* mutations when only *ABCA4* has been screened. Though rare, these cases support the implementation of exome sequencing to detect potentially confounding genetic mutations as well as enable the study of the effect of multi-gene mutation combinations on disease progression and severity.

Mutations in *CRB1* have been associated with a wide array of retinal dystrophies, including retinitis pigmentosa and Leber congenital amaurosis [41]. Here we report the first finding of a heterozygous nonsense mutation in *CRB1* in an individual with macular degeneration. Given that *CRB1* is involved in normal retinal development, this variant is an intriguing functional candidate. A thickening of the fovea (without the cavitations associated with macular edema) observable by OCT in this participant is consistent with other cases of *CRB1*-related disease. In the absence of a functional validation of the p.K801X variant or analysis of additional cases with nonsense variants in *CRB1*, it is not possible at present to confidently assign a molecular diagnosis to this case and we thus categorize this variant as being of unknown significance. This inability to causally link a suspect variant with phenotype is a significant limitation of the technique, and will likely be typical of early clinical WES efforts.

While it is attractive to attempt to correlate clinical and phenotypic information with our newly acquired genetic data, sample size is a significant limitation. For example, one might hypothesize that individuals with *PRPH2* dysfunction would have abnormal cone/rod responses due to its restricted expression to the outer disc segment of photoreceptors [42]. However, given the range of variability across Stargardt cases and the small number of *PRPH2* positive cases, it is not possible to assess any such correlation at present. Collaboration and data sharing amongst multiple STGD research groups is needed to sufficiently increase the sample size to evaluate any genotype-phenotype correlations.

In this study, we suffer from the unavailability of complete family history information and genetic material from related individuals, particularly for participants with variants outside of *ABCA4*. These limitations are common in clinical practice. While both history and DNA would provide critical insight into mode of inheritance and shed light on the functional consequences of observed genetic variants, we identified a clear molecular basis of disease in six out of nine cases.

Despite near total coverage of most genes, including both *ABCA4* and *PRPH2* in all nine cases, several of our cases elude a definitive molecular diagnosis. One case has only a single *ABCA4* mutation, one has a variant of unknown significance in *CBR1* and one sample has no demonstrable mutations in any gene with known association to retinal or macular degeneration. Careful manual inspection of genes carrying potential compound heterozygous, homozygous, and protein-truncating variants in these individuals did not yield any strong functional candidates for novel STGD disease genes. It is now generally accepted that every individual carries a significant burden of potentially pathogenic DNA variants within the known exome [43], and STGD cases are no different in this regard. Which, if any, such variants in genes not currently associated with retinopathy in our sample contribute to disease onset and progression cannot be assessed at this time, nor can we rigorously address the potential of other genetic variants acting in a dominant fashion or in a digenic manner. The exome data provides an opportunity to make a concerted effort to re-analyze these data as new disease genes and genetic modifiers are implicated in retinal and macular dystrophies. In the meantime, several of our cases elude molecular diagnosis. While such cases represent a small proportion of the overall STGD participant population, they may eventually provide invaluable insight into the pathogenesis of STGD and the biology of the retina.

The disease phenotype of STGD can be quite variable, even amongst individuals carrying identical or putatively similar *ABCA4* mutations. Age of onset, electroretinogram response, disease progression and disease severity are all clinically relevant parameters of phenotypic variability in these participants. While some genotype-phenotype correlations have been found for population-specific *ABCA4* mutations [11], the majority of this variability remains unexplained. One major benefit of exome sequencing over targeted *ABCA4* mutation screening is the potential for identifying disease-modifying alleles in other genes. If approached properly, clinical exome sequencing can enable such studies as a byproduct of providing highly sensitive molecular testing.

Knowledge of genome-wide functional variation may be particularly critical to enrollment of participants in future clinical trials. Restoration or replacement of ABCA4 protein function would likely be insufficient for a participant harboring disease-causing mutations in additional genes, such as seen with *PRPH2* in this study. Thus the positive identification of two mutations in *ABCA4* may not be a proper endpoint for molecular diagnosis in STGD. In contrast, exome sequencing can help place STGD participants into genomic context as it identifies both common and rare functional genetic variation.

Clinicians now have a multitude of options available to them for STGD mutation screening. Targeted approaches continue to grow in sensitivity and shrink in cost [20], but they will always be fundamentally limited to specific genes or alleles. From these small-scale results, we find very strong potential for STGD molecular diagnosis by exome sequencing. Presently, an approach following up array-based *ABCA4* mutation screening with exome sequencing is a prudent and cost-effective alternative to direct re-sequencing. In the near future, exome sequencing and indeed whole genome sequencing will likely be sufficiently inexpensive (and potentially covered by health insurance), eliminating the need for STGD-specific mutation screening.

While important issues such as off-target findings and variants of unknown significance remain highly controversial in the field of genomic diagnostics, harnessing WES technology to provide molecular diagnosis for a specific subset of genes – essentially performing gene panels by exome analysis – largely circumvents these issues. If participants are properly consented, willing to participate, and educated properly about the risks, benefits, and scope of such testing, clinical molecular diagnosis and research can and should be pursued simultaneously. With sufficient sample sizes, a deeper understanding of locus heterogeneity and perhaps even modifier genes becomes possible. This interplay between clinical testing and research should provide better patient care and pave the way toward an improved understanding of Stargardt Disease and macular degeneration in general.

## Conclusions

Genomic analysis is the most direct means to resolve the clinical overlap of inherited early onset macular degeneration disorders. Based upon the striking level of success in our most difficult to classify cases, clinical exome sequencing should be considered as a potential front line diagnostic tool for retinal and macular dystrophies with atypical presentations, as a follow-up after negative or unclear results from initial screening by targeted mutation analysis, Additionally, WES may prove crucial in ruling out rare mutations in genes that may confound the determination of causality before proceeding to gene-targeted therapies.

## Abbreviations

STGD: Stargardt Disease; WES: whole exome sequencing; OD: right eye; OS: left eye; OCT: Optical coherence tomography; ERG: electroretinogram; Mb: megabases.

## Competing interests

The authors claim no financial conflicts or competing interests relevant to this study.

## Authors’ contributions

SPS analyzed the exome sequence data, and wrote and prepared the manuscript. YQG performed dideoxy DNA sequencing and analyzed the results. AM provided genetic counseling and pedigree analysis, and aided in the phenotypic assessment of participants. CO recruited study participants and aided in phenotypic assessment. ZC directed the preparation and high throughput sequencing of exomic libraries. SFN provided the means, expertise, and computational resources to perform and analyze exome sequencing data. SN performed and analyzed results of electroretinogram measurements. DBF was a co-investigator, involved in sample recruitment, conceptualization, and all dideoxy sequencing analysis. MBG, the principal investigator, conceived this project and contributed to all aspects of this work including clinical examination of participants, and interpretation of results. All authors read and approved the final manuscript.

## Pre-publication history

The pre-publication history for this paper can be accessed here:

http://www.biomedcentral.com/1471-2350/13/67/prepub

## Supplementary Material

Additional file 1 **Figure S1**. Autofluorescence (A,B) and infrared (C,D) imaging of participant STGD-01. **Figure S2**. Fundus photos of participant STGD-02. **Figure S3**. Autofluorescence (AF) imaging and optical coherence tomography (OCT) images for participant STGD-03. 50° AF image of OD (A) and OS (B) shows geographic atrophy, discrete autofluorescent flecks, and peripapillary sparing in both eyes. OCT of OD (C) shows severe retinal edema. **Figure S4**. Autofluorescent imaging of participant STGD-04. 30° AF image of OD (A) shows “stellate” pattern dystrophy. Pattern less clear in OS (B). **Figure S5**. Autofluorescent imaging of participant STGD-05. 50° AF imaging of both OD (A) and OS (B) shows possible “stellate” pattern dystrophy. **Figure S6**. Red-free fundus images of participant STGD-06. 30° fundus images of both OD (A) and OS (B) show wide-spread geographic atrophy of the macula with mild peripapillary sparing. **Figure S7**. Color fundus images of participant STGD-07. 50° color fundus images from both OD (A) and OS (B) show geographic atrophy with peripapillary sparing. **Figure S8**. Autofluorescence and optical coherence tomography images for participant STGD-08. 50° AF shows irregular geographic atrophy without peripapillary sparing or autofluorescent flecks in both OD (A) and OS (B). OCT shows an irregularly thickened contour of the retina in both OD (C) and OS (D). **Figure S9**. Fundus photos of OD (A) and OS (B) for participant STGD-08. Clear “horse-shoe” pattern of atrophy is observable in both eyes. Click here for file

Additional file 2 **Table S1.** Whole Exome Sequencing Details Click here for file

Additional file 3 **Figure S1.** Autofluorescence (A,B) and infrared (C,D) imaging of participant STGD-01. Click here for file

Additional file 4 **Figure S2.** Fundus photos of participant STGD-02. Click here for file

Additional file 5 **Figure S3.** Autofluorescence (AF) imaging and optical coherence tomography (OCT) images for participant STGD-03. 50° AF image of OD (A) and OS (B) shows geographic atrophy, discrete autofluorescent flecks, and peripapillary sparing in both eyes. OCT of OD (C) shows severe retinal edema. Click here for file

Additional file 6 **Figure S4.** Autofluorescent imaging of participant STGD-04. 30° AF image of OD (A) shows “stellate” pattern dystrophy. Pattern less clear in OS (B). Click here for file

Additional file 7 **Figure S5.** Autofluorescent imaging of participant STGD-05. 50° AF imaging of both OD (A) and OS (B) shows possible “stellate” pattern dystrophy. Click here for file

Additional file 8 **Figure S7.** Color fundus images of participant STGD-07. 50° color fundus images from both OD (A) and OS (B) show geographic atrophy with peripapillary sparing. Click here for file

Additional file 9 **Figure S6.** Red-free fundus images of participant STGD-06. 30° fundus images of both OD (A) and OS (B) show wide-spread geographic atrophy of the macula with mild peripapillary sparing. Click here for file

Additional file 10 **Figure S8.** Autofluorescence and optical coherence tomography images for participant STGD-08. 50° AF shows irregular geographic atrophy without peripapillary sparing or autofluorescent flecks in both OD (A) and OS (B). OCT shows an irregularly thickened contour of the retina in both OD (C) and OS (D). Click here for file

Additional file 11 **Figure S9.** Fundus photos of OD (A) and OS (B) for participant STGD-08. Clear “horse-shoe” pattern of atrophy is observable in both eyes. Click here for file
