# Supplementary material for: Molecular diagnosis of putative Stargardt disease probands by exome sequencing
Source: BMC Med Genet. 2012 Aug 3;13:67. doi: 10.1186/1471-2350-13-67 (PMC3459799; doi:10.1186/1471-2350-13-67)
Supplement: Additional file 1 — Figure S1. Autofluorescence (A,B) and infrared (C,D) imaging of participant STGD-01. Figure S2. Fundus photos of participant STGD-02. Figure S3. Autofluorescence (AF) imaging and optical coherence tomography (OCT) images for participant STGD-03. 50° AF image of OD (A) and OS (B) shows geographic atrophy, discrete autofluorescent flecks, and peripapillary sparing in both eyes. OCT of OD (C) shows severe retinal edema. Figure S4. Autofluorescent imaging of participant STGD-04. 30° AF image of OD (A) shows “stellate” pattern dystrophy. Pattern less clear in OS (B). Figure S5. Autofluorescent imaging of participant STGD-05. 50° AF imaging of both OD (A) and OS (B) shows possible “stellate” pattern dystrophy. Figure S6. Red-free fundus images of participant STGD-06. 30° fundus images of both OD (A) and OS (B) show wide-spread geographic atrophy of the macula with mild peripapillary sparing. Figure S7. Color fundus images of participant STGD-07. 50° color fundus images from both OD (A) and OS (B) show geographic atrophy with peripapillary sparing. Figure S8. Autofluorescence and optical coherence tomography images for participant STGD-08. 50° AF shows irregular geographic atrophy without peripapillary sparing or autofluorescent flecks in both OD (A) and OS (B). OCT shows an irregularly thickened contour of the retina in both OD (C) and OS (D). Figure S9. Fundus photos of OD (A) and OS (B) for participant STGD-08. Clear “horse-shoe” pattern of atrophy is observable in both eyes. [file 1471-2350-13-67-S1.doc]

**Supplemental Figure Legends**

Supplemental Figure 1. Autofluorescence (A,B) and infrared (C,D) imaging of participant STGD-01.

Supplemental Figure 2. Fundus photos of participant STGD-02.

Supplemental Figure 3. Autofluorescence (AF) imaging and optical coherence tomography (OCT) images for participant STGD-03. 50° AF image of OD (A) and OS (B) shows geographic atrophy, discrete autofluorescent flecks, and peripapillary sparing in both eyes. OCT of OD (C) shows severe retinal edema.

Supplemental Figure 4. Autofluorescent imaging of participant STGD-04. 30° AF image of OD (A) shows “stellate” pattern dystrophy. Pattern less clear in OS (B).

Supplemental Figure 5. Autofluorescent imaging of participant STGD-05. 50° AF imaging of both OD (A) and OS (B) shows possible “stellate” pattern dystrophy.

Supplemental Figure 6. Red-free fundus images of participant STGD-06. 30° fundus images of both OD (A) and OS (B) show wide-spread geographic atrophy of the macula with mild peripapillary sparing.

Supplemental Figure 7. Color fundus images of participant STGD-07. 50° color fundus images from both OD (A) and OS (B) show geographic atrophy with peripapillary sparing.

Supplemental Figure 8. Autofluorescence and optical coherence tomography images for participant STGD-08. 50° AF shows irregular geographic atrophy without peripapillary sparing or autofluorescent flecks in both OD (A) and OS (B). OCT shows an irregularly thickened contour of the retina in both OD (C) and OS (D).

Supplemental Figure 9. Fundus photos of OD (A) and OS (B) for participant STGD-08. Clear “horse-shoe” pattern of atrophy is observable in both eyes.
