# Supplementary material for: Molecular diagnosis of putative Stargardt disease probands by exome sequencing
Source: BMC Med Genet. 2012 Aug 3;13:67. doi: 10.1186/1471-2350-13-67 (PMC3459799; doi:10.1186/1471-2350-13-67)
Supplement: Additional file 2 — Table S1. Whole Exome Sequencing Details [file 1471-2350-13-67-S2.doc]

Supplemental Table 1. Whole Exome Sequencing Details

| Patient ID | Mean  Coverage | Sequencer | Mean | Total Variants  (indels) | Novel Variants (percent) | *ABCA4*  Min | *ABCA4*  % ≥ 8x | *ABCA4*  % ≥20x |
| --- | --- | --- | --- | --- | --- | --- | --- | --- |
| STGD-01 | 104 | HiSeq2000 | 134.8 | 206,211  (17,907) | 7,626 (3.7%) | 15 | 100 | 99.6 |
| STGD-02 | 107 | HiSeq2000 | 133.1 | 176,999  (15,836) | 6,187 (3.5%) | 14 | 100 | 99.8 |
| STGD-03 | 121 | HiSeq2000 | 140.2 | 245,904  (21,178) | 14,052 (5.7%) | 8 | 100 | 99.5 |
| STGD-04 | 105 | HiSeq2000 | 119.6 | 211,989  (18,330) | 6,351 (3%) | 14 | 100 | 99.6 |
| STGD-05 | 103 | HiSeq2000 | 114.9 | 215,358  (18,613) | 6,702 (3.1%) | 12 | 100 | 98.6 |
| STGD-06 | 44 | GAIIx | 55.9 | 104,250  (8,641) | 5,793 (5.6%) | 1 | 99.1 | 87.6 |
| STGD-07 | 48 | GAIIx | 56.3 | 95,899  (8,416) | 3,511 (3.7%) | 3 | 98.2 | 90.0 |
| STGD-08 | 46 | GAIIx | 57.9 | 95,553  (8,224) | 3,196 (3.3%) | 3 | 99.5 | 90.7 |
| STGD-09 | 51 | GAIIx | 60.7 | 107,035  (9,242) | 3,958 (3.7%) | 2 | 99.4 | 90.5 |

Mean Coverage is calculated as the average read depth across all target loci provided by manufacturer. The number of sequence reads mapping to all 6,822 protein-coding base pairs in the *ABCA4* gene were calculated for each sample. The mean read depth, minimum read depth (“Min”) are reported, as well as the percentage of bases covered by at least eight reads (the minimum required for variant calling) and twenty reads (%≥8x and %≥20x respectively).
