# Supplementary figures and images for: Molecular diagnosis of putative Stargardt disease probands by exome sequencing
Source: BMC Med Genet. 2012 Aug 3;13:67. doi: 10.1186/1471-2350-13-67 (PMC3459799; doi:10.1186/1471-2350-13-67)

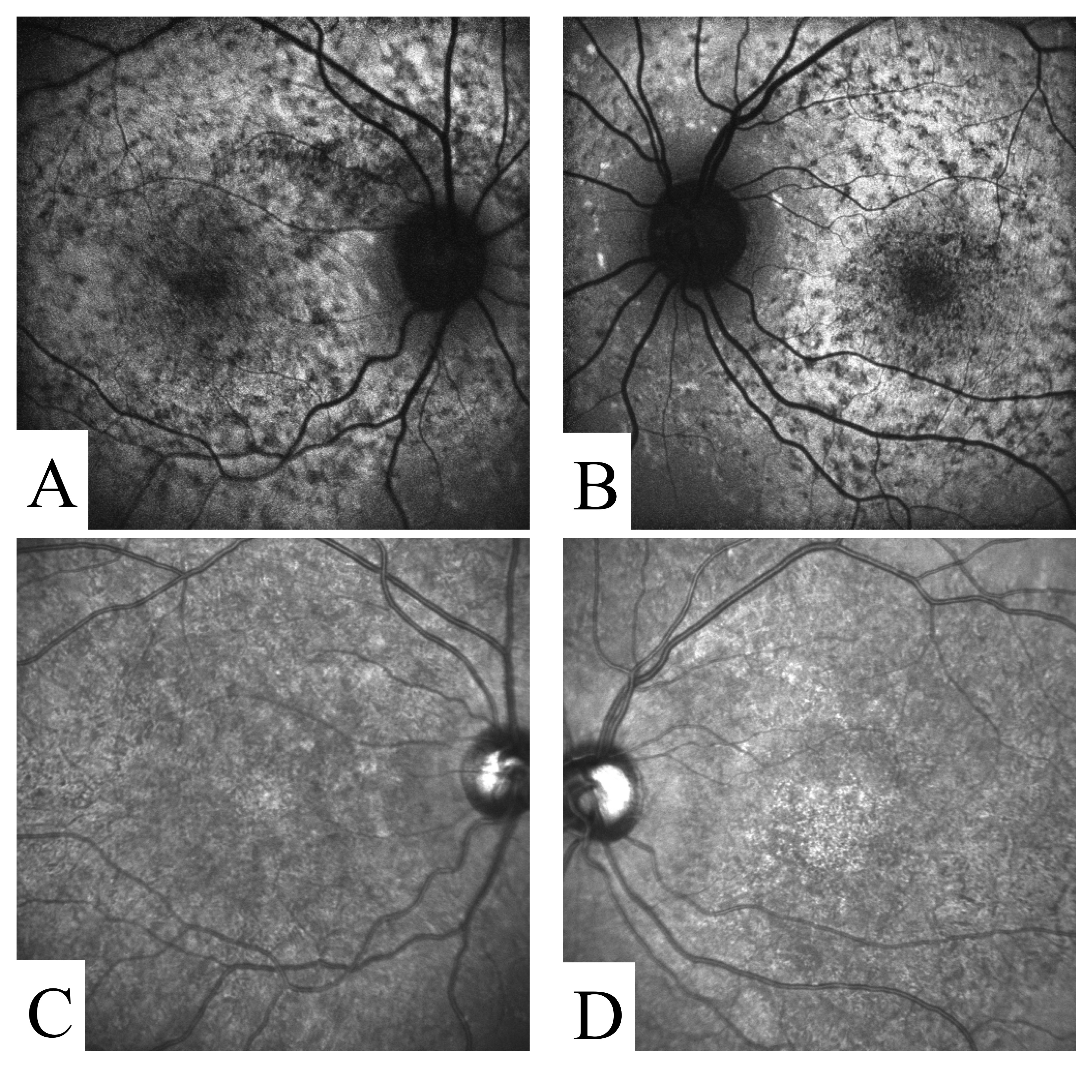

Supplement: Additional file 3 — Figure S1. Autofluorescence (A,B) and infrared (C,D) imaging of participant STGD-01. [file 1471-2350-13-67-S3.jpeg]

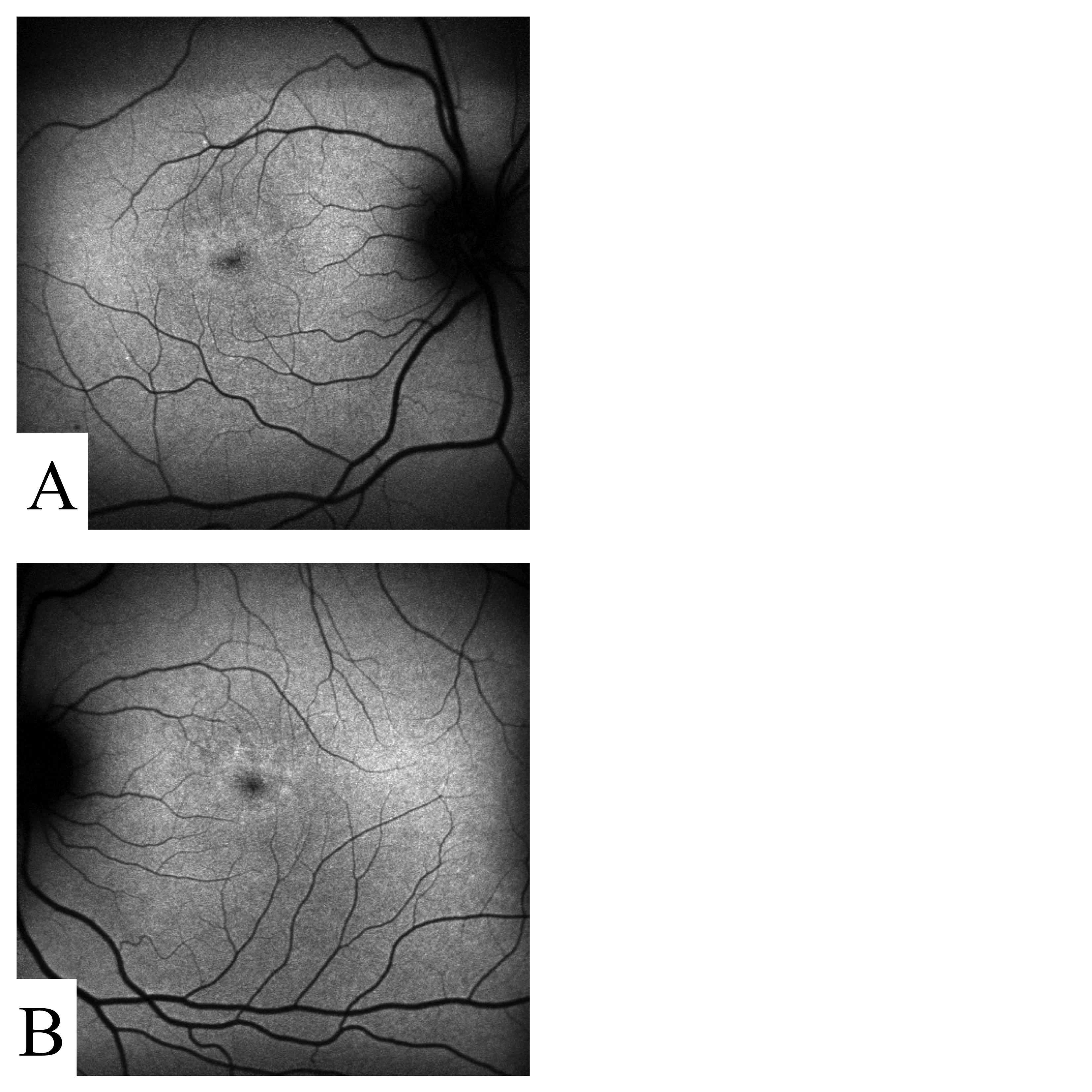

Supplement: Additional file 4 — Figure S2. Fundus photos of participant STGD-02. [file 1471-2350-13-67-S4.jpeg]

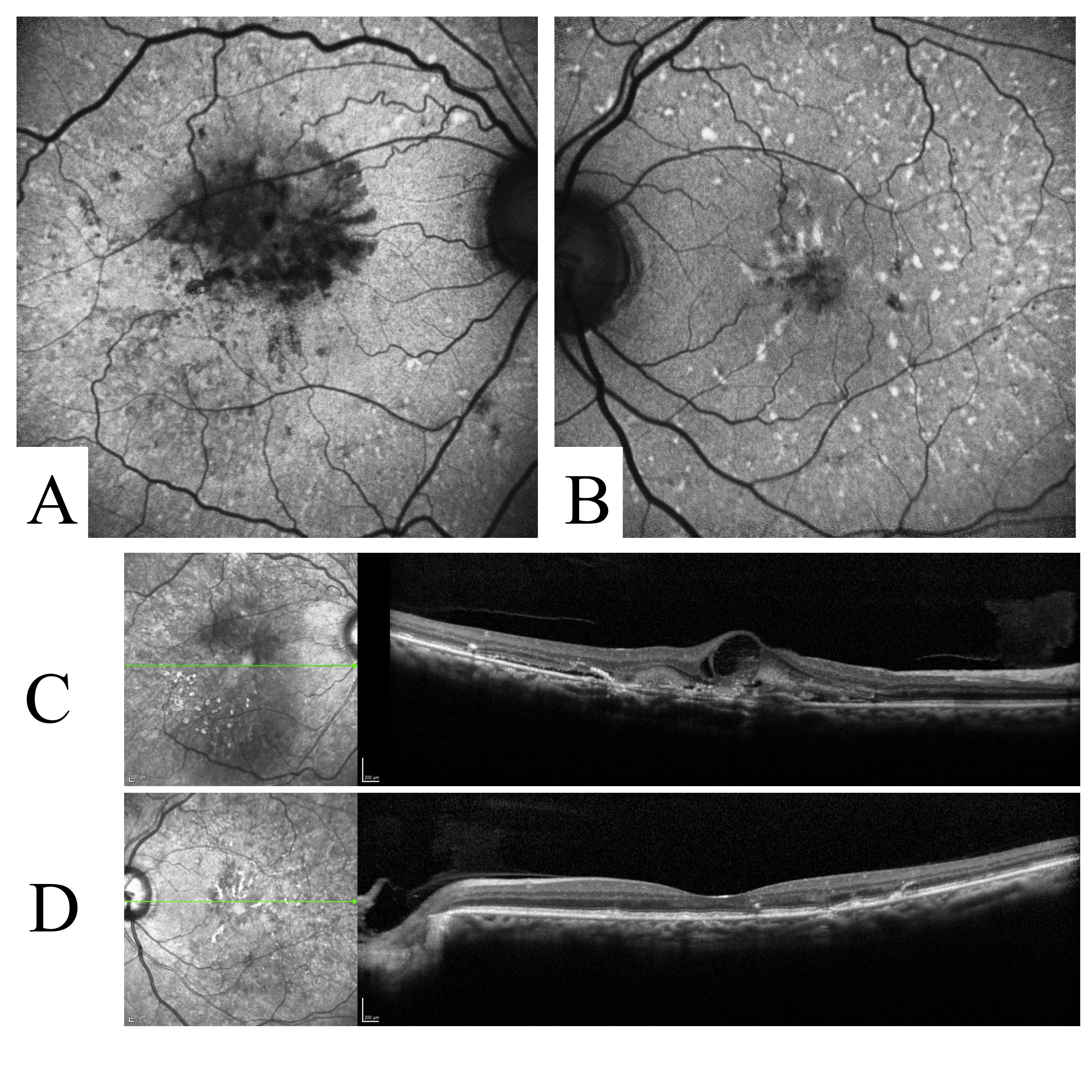

Supplement: Additional file 5 — Figure S3. Autofluorescence (AF) imaging and optical coherence tomography (OCT) images for participant STGD-03. 50° AF image of OD (A) and OS (B) shows geographic atrophy, discrete autofluorescent flecks, and peripapillary sparing in both eyes. OCT of OD (C) shows severe retinal edema. [file 1471-2350-13-67-S5.jpeg]

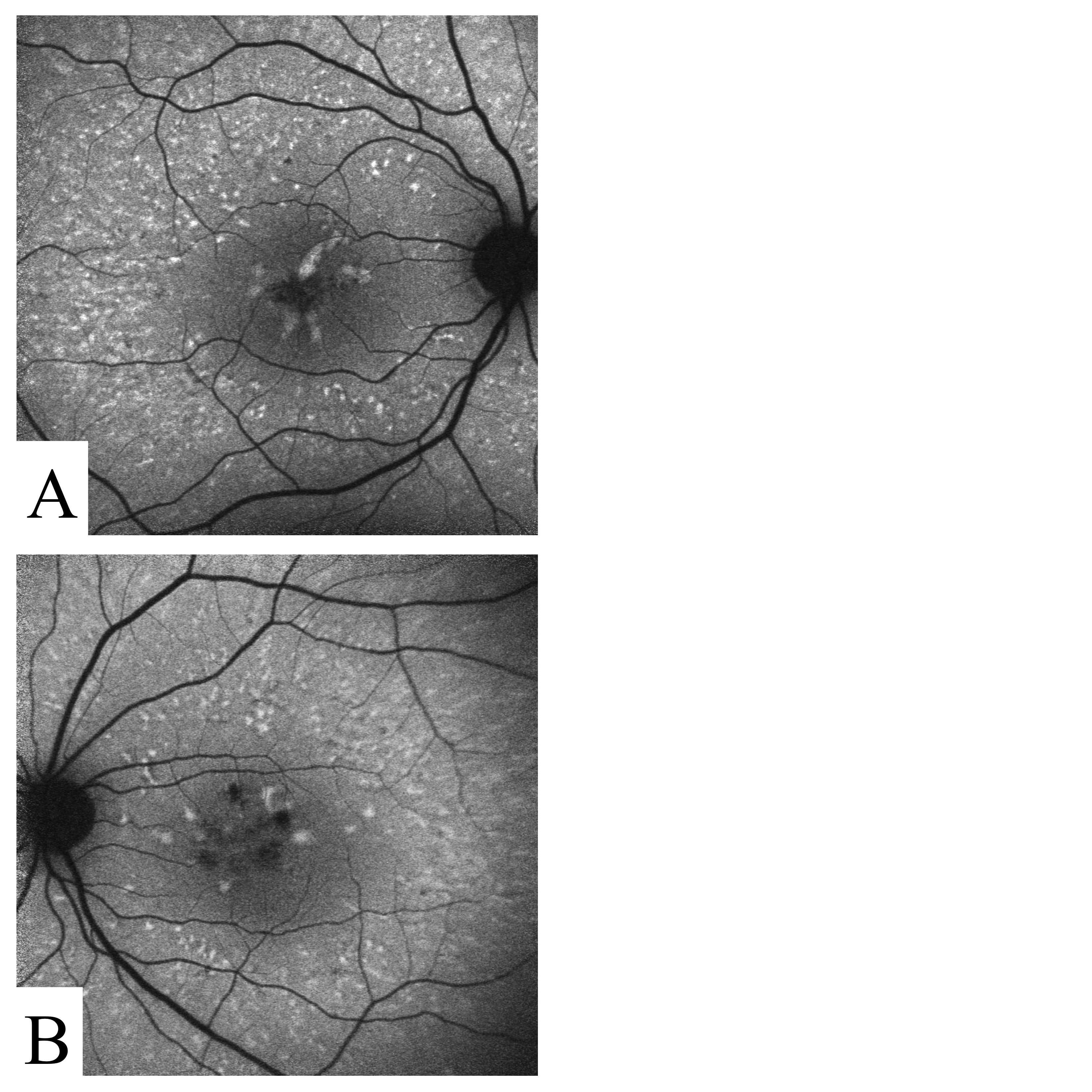

Supplement: Additional file 6 — Figure S4. Autofluorescent imaging of participant STGD-04. 30° AF image of OD (A) shows “stellate” pattern dystrophy. Pattern less clear in OS (B). [file 1471-2350-13-67-S6.jpeg]

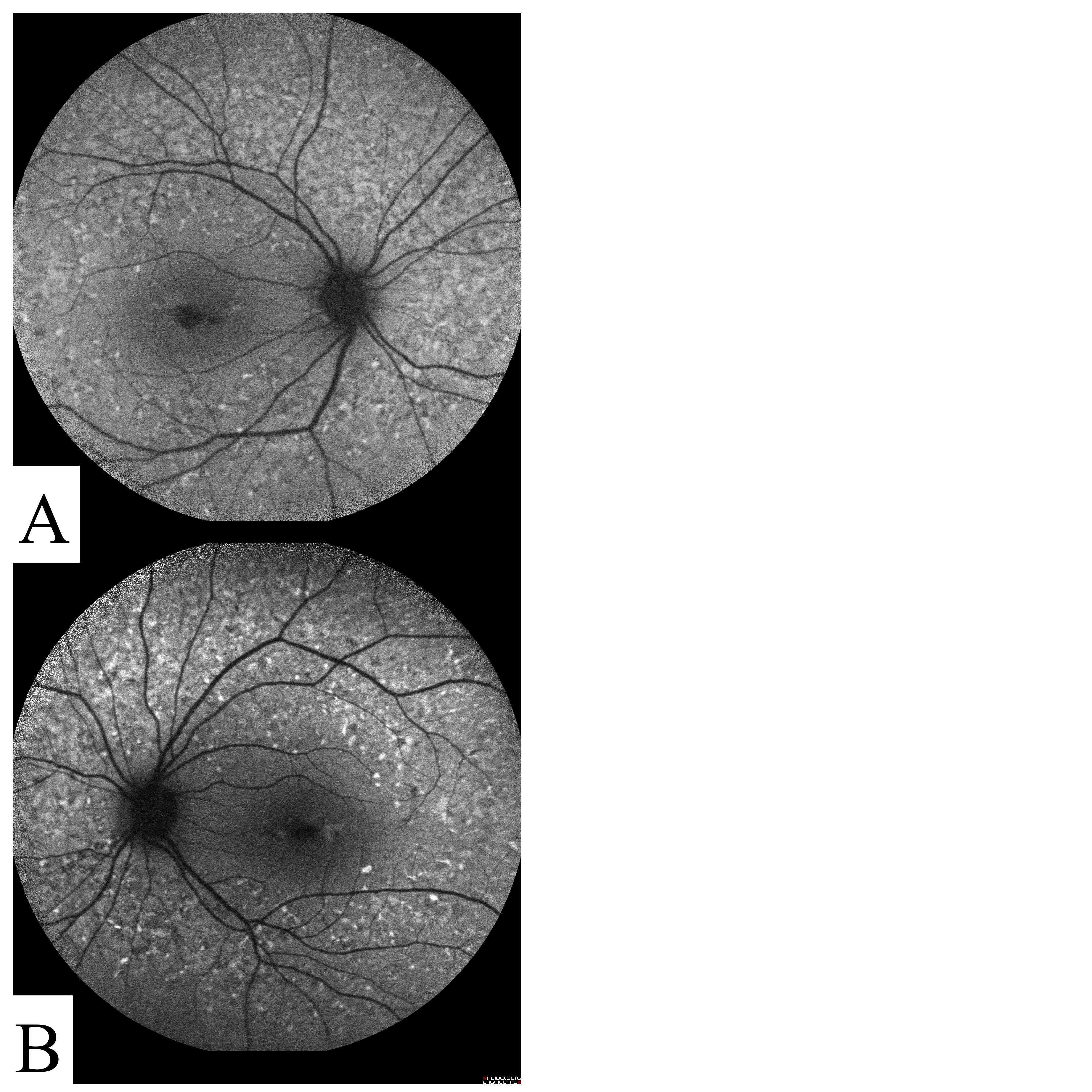

Supplement: Additional file 7 — Figure S5. Autofluorescent imaging of participant STGD-05. 50° AF imaging of both OD (A) and OS (B) shows possible “stellate” pattern dystrophy. [file 1471-2350-13-67-S7.jpeg]

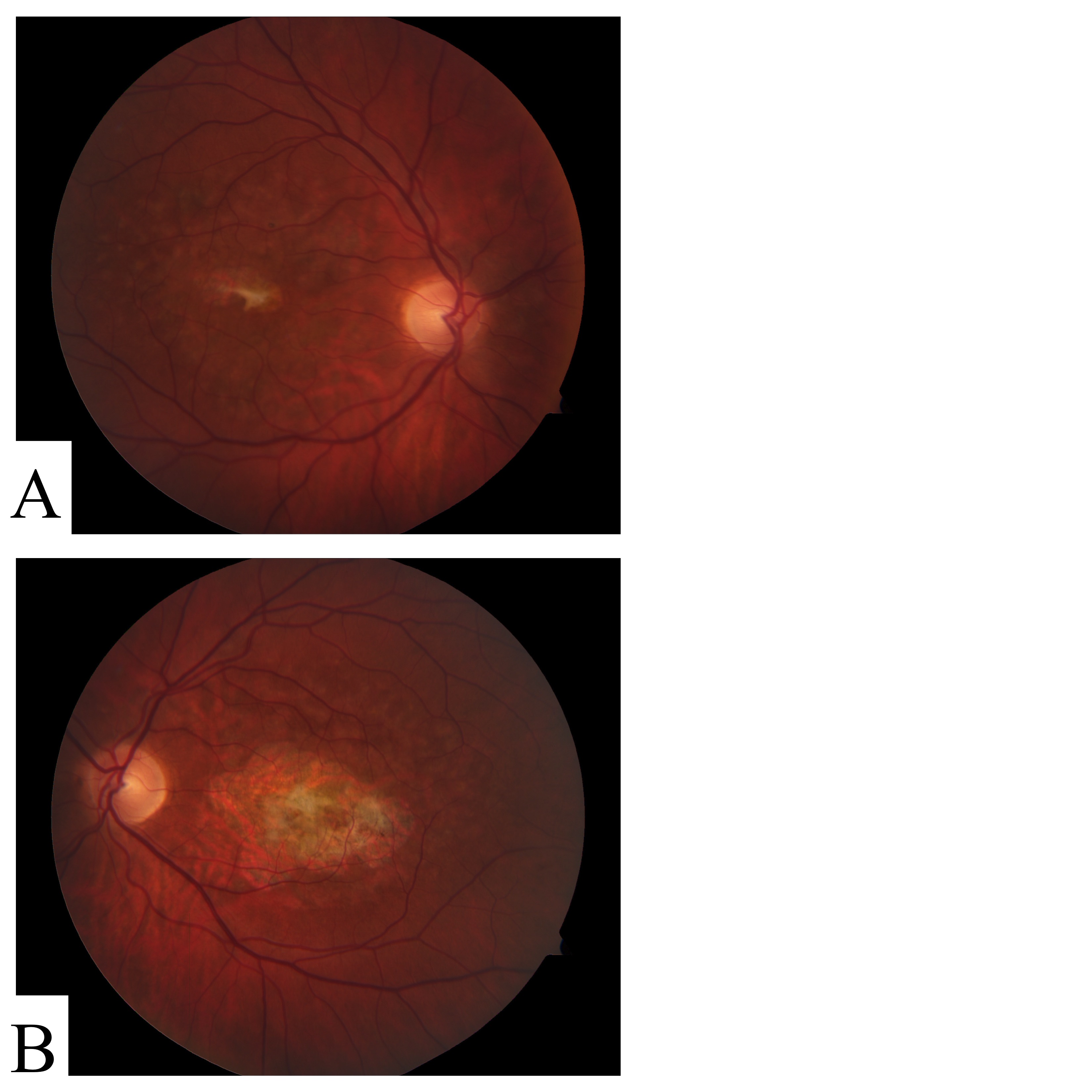

Supplement: Additional file 8 — Figure S7. Color fundus images of participant STGD-07. 50° color fundus images from both OD (A) and OS (B) show geographic atrophy with peripapillary sparing. [file 1471-2350-13-67-S8.jpeg]

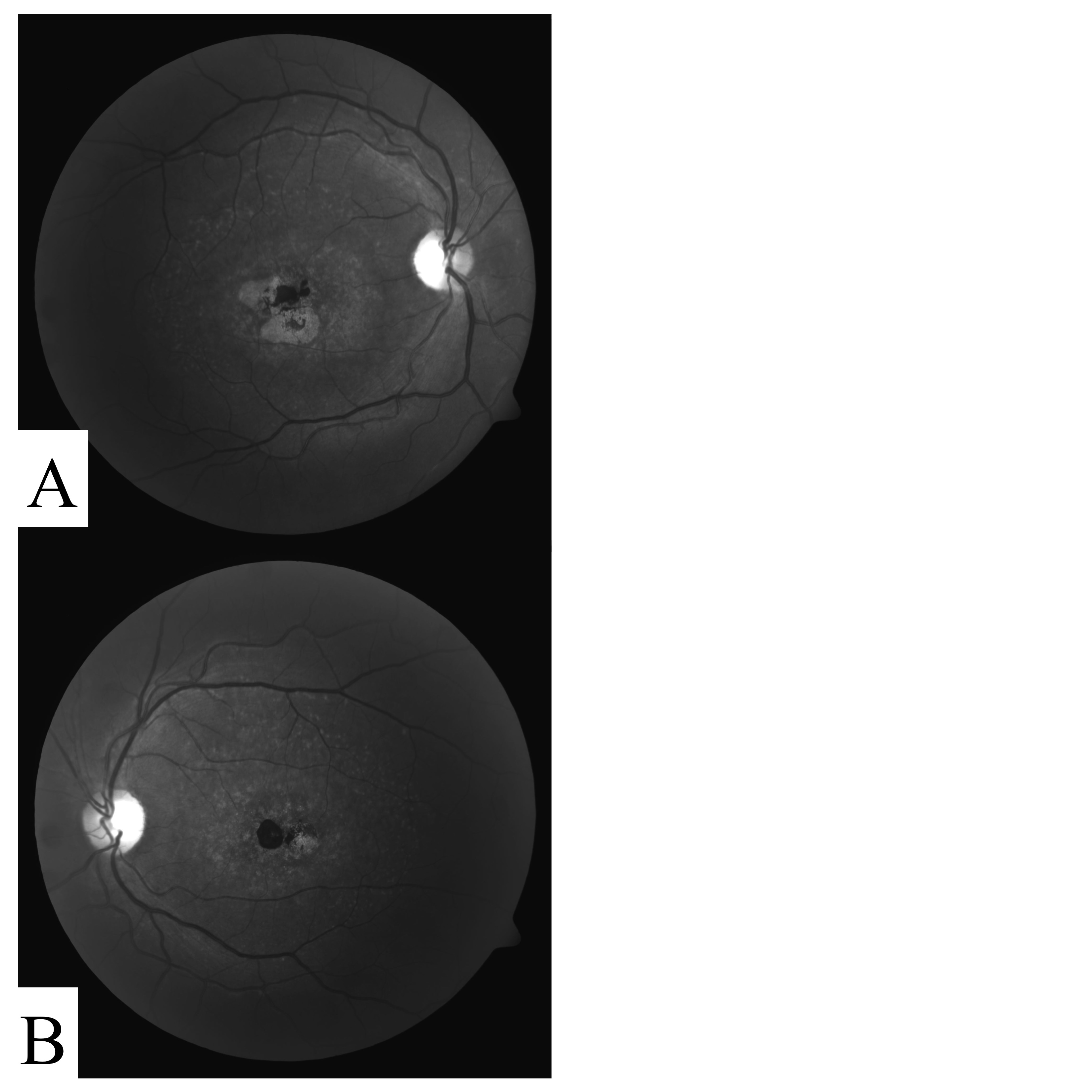

Supplement: Additional file 9 — Figure S6. Red-free fundus images of participant STGD-06. 30° fundus images of both OD (A) and OS (B) show wide-spread geographic atrophy of the macula with mild peripapillary sparing. [file 1471-2350-13-67-S9.jpeg]

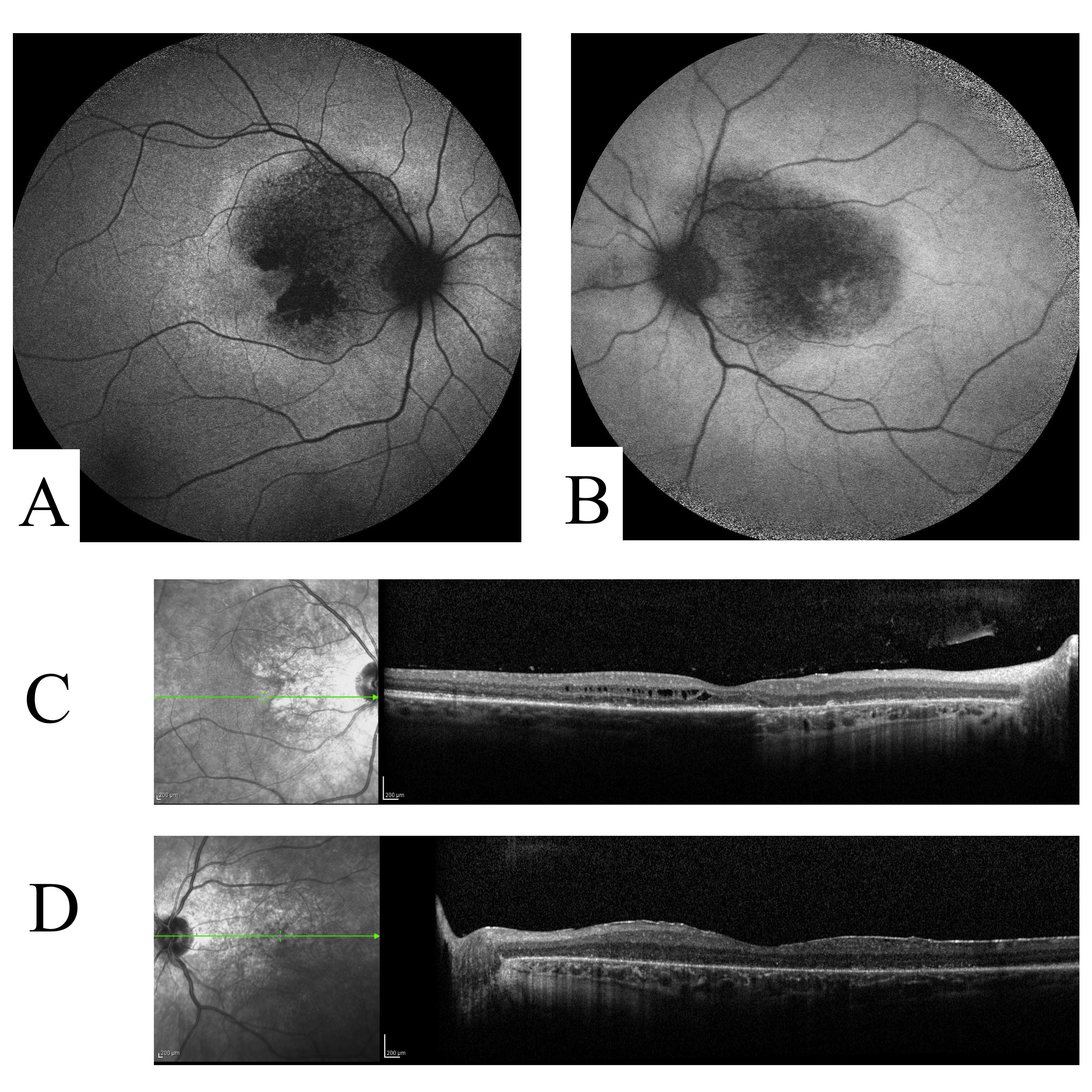

Supplement: Additional file 10 — Figure S8. Autofluorescence and optical coherence tomography images for participant STGD-08. 50° AF shows irregular geographic atrophy without peripapillary sparing or autofluorescent flecks in both OD (A) and OS (B). OCT shows an irregularly thickened contour of the retina in both OD (C) and OS (D). [file 1471-2350-13-67-S10.png]

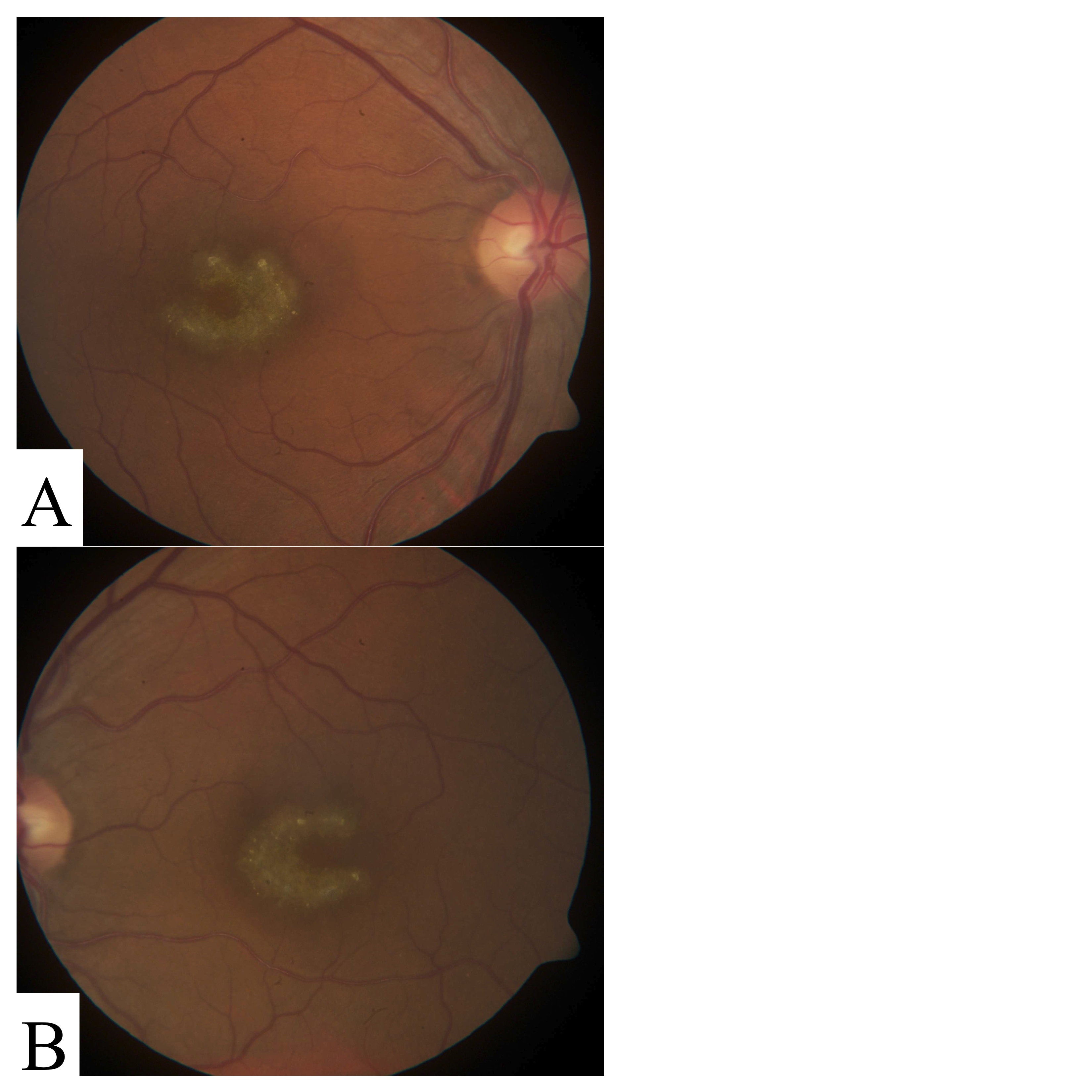

Supplement: Additional file 11 — Figure S9. Fundus photos of OD (A) and OS (B) for participant STGD-08. Clear “horse-shoe” pattern of atrophy is observable in both eyes. [file 1471-2350-13-67-S11.jpeg]
